# Plant Transformation States and Exposure Architecture: A Pharmacokinetic Framework for Plant-Derived Compounds in Bone Remodeling

**DOI:** 10.3390/plants15101541

**Published:** 2026-05-18

**Authors:** Sara Khaleel, Tariq Al-Qirim, Ala A. Alhusban, Talal Aburjai, Thaqif El Khassawna

**Affiliations:** 1 Department of Pharmacy, Faculty of Pharmacy, Al-Zaytoonah University of Jordan, Amman 11733, Jordan; s.malkawi@zuj.edu.jo (S.K.); tariq.qirim@zuj.edu.jo (T.A.-Q.); ala.alhusban@zuj.edu.jo (A.A.A.); 2 Department of Pharmaceutical Sciences, School of Pharmacy, The University of Jordan, Amman 11942, Jordan; 3 Experimental Trauma Surgery, Faculty of Medicine, Justus-Liebig-University of Giessen, 35392 Giessen, Germany

**Keywords:** bone remodeling, exposure architecture, plant-derived compounds, pharmacokinetics, bioavailability, plant matrix, microbiota, nanodelivery systems

## Abstract

Plant-derived compounds exhibit well-documented osteogenic and anti-resorptive activities; however, their translation into consistent skeletal benefits remains limited. This review proposes a transformation-state-dependent framework in which the efficacy of plant-based interventions is interpreted through the exposure architectures they generate rather than solely through intrinsic molecular activity. By integrating plant matrix organization, gastrointestinal processing, microbial biotransformation, and formulation-driven pharmacokinetics with the temporal dynamics of bone remodeling, the review addresses a critical gap in the current literature, which largely evaluates phytochemicals independent of their delivery context. Across a continuum ranging from intact plant matrices to isolated compounds and advanced delivery systems, distinct pharmacokinetic regimes emerge, characterized by differences in release kinetics, metabolic transformation, systemic persistence, and target-site exposure. Representative interventions showing promising pharmacokinetic and skeletal findings include curcumin phytosome systems, resveratrol nanoformulations, icariin-loaded delivery platforms, and matrix-associated polyphenol systems capable of promoting sustained or metabolite-mediated exposure. Evidence indicates that sustained, metabolite-mediated exposure profiles are more compatible with the prolonged, cumulative nature of bone remodeling, whereas transient exposure often limits efficacy despite mechanistic activity. Formulation strategies, including phospholipid complexes, bioenhancers, and nano- or vesicle-based systems, can partially overcome these limitations by modulating exposure behavior. By reframing plant-based interventions as dynamic exposure systems, this framework provides a unifying basis for interpreting variability across studies and offers a rational foundation for designing strategies that align pharmacokinetic behavior with skeletal biology, thereby improving translational potential.

## 1. Introduction

Bone disorders, including osteopenia, osteoporosis, and inflammation-associated bone loss, remain difficult to treat despite extensive advances in understanding the molecular regulation of skeletal homeostasis [1,2].

Conventional pharmacological management of bone loss relies mainly on antiresorptive agents, including bisphosphonates and denosumab, and anabolic or bone-forming agents such as teriparatide, abaloparatide, and romosozumab [3,4]. Although these therapies are clinically effective, their long-term use remains associated with several treatment-specific limitations. Bisphosphonates, for example, may cause gastrointestinal intolerance and exhibit prolonged skeletal retention following long-term administration. In addition, extended antiresorptive therapy has been associated with rare but clinically important adverse events, including atypical femoral fractures and osteonecrosis of the jaw [4]. Denosumab therapy presents a different pharmacological challenge because treatment discontinuation may lead to rebound increases in bone turnover and elevated vertebral fracture risk [5]. Similarly, parathyroid hormone analogues are constrained by restricted treatment duration, limiting their long-term applicability in chronic skeletal disorders.

Furthermore, although romosozumab demonstrates strong anabolic and antiresorptive efficacy, its use requires cardiovascular caution in selected patient populations [4]. Collectively, these limitations support continued exploration of complementary skeletal strategies, including plant-derived interventions capable of providing sustained and biologically compatible exposure profiles.

Numerous plant-derived compounds and extracts exhibit anti-resorptive, osteogenic, and anti-inflammatory activities in experimental systems through modulation of key signaling pathways, including Wnt/β-catenin, phosphoinositide 3-kinase/protein kinase B (PI3K/Akt), nuclear factor kappa B (NF-κB), and receptor activator of nuclear factor kappa B ligand (RANKL)-mediated osteoclastogenesis; however, their clinical translation remains inconsistent and often limited [6]. This discrepancy suggests that the principal limitation in plant-based bone therapeutics lies not in insufficient molecular activity—well established through their regulation of osteoblastogenesis and osteoclastogenesis—but in pharmacokinetic and translational constraints. These include limited bioavailability, poor solubility, rapid metabolism and elimination, dose-dependent responses, and inter-individual variability in absorption and metabolic processing, which collectively generate heterogeneous systemic exposure profiles and hinder reproducible clinical efficacy [7].

Bone can therefore be viewed as a pharmacokinetically constrained biological system, in which therapeutic efficacy depends primarily on exposure persistence across remodeling-relevant timescales rather than on peak concentration or intrinsic molecular potency alone.

In this review, “exposure architecture” refers to the integrated pharmacokinetic pattern generated by a plant-derived intervention, including the timing of release, site and rate of absorption, systemic exposure magnitude, circulating half-life, metabolic transformation, microbiota-derived metabolite formation, and persistence of biologically relevant parent compounds or metabolites. This term is therefore broader than bioavailability alone: whereas bioavailability describes the extent of systemic entry, exposure architecture describes how exposure is temporally and metabolically organized in relation to the prolonged biological requirements of bone remodeling.

**Figure 1:**
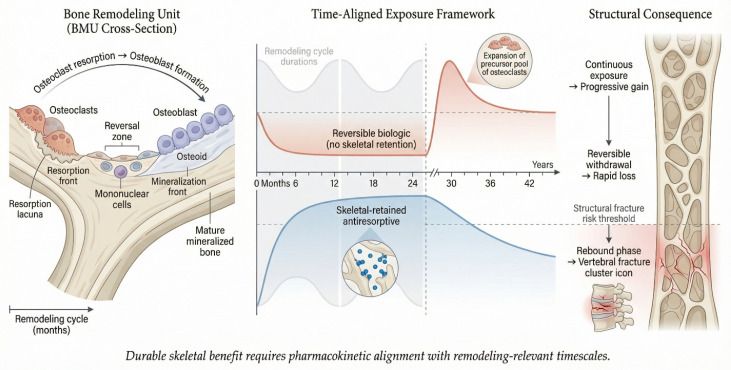
Schematic representation of the bone remodeling unit (**left**), illustrating coordinated osteoclastic resorption and osteoblastic formation within a temporally extended cycle. The (**central**) panel depicts how differing exposure profiles—transient versus sustained, with or without skeletal retention—interact with remodeling timescales to determine biologic persistence. The (**right**) panel highlights the resulting structural consequences, including progressive gain with continuous exposure, rapid loss following withdrawal of reversible agents, and rebound-associated fracture risk. Collectively, the figure emphasizes that durable skeletal efficacy requires alignment of pharmacokinetic exposure with remodeling-relevant temporal scales. Generated using Nano Banana Pro (https://picir.ai/m/nano-banana/nano-banana-pro, accessed on 1 April 2026).

Bone remodeling occurs within basic multicellular units (BMUs), where tightly coordinated and overlapping phases of activation, osteoclastic resorption, reversal, osteoblastic formation, and termination proceed over a cycle lasting approximately 120–200 days in humans [8]. As illustrated in Figure 1, these processes represent temporally integrated biological programs rather than rapid signaling events.

Consequently, meaningful modification of bone mass or microarchitecture requires sustained modulation of remodeling pathways across successive cycles operating over weeks to months within basic multicellular units. Bone remodeling is a continuous, tightly coupled process in which osteoclastic resorption and osteoblastic formation are coordinated through local and systemic signaling [9]. This temporal constraint is particularly evident in osteoclastogenesis, which requires sustained RANKL signaling to maintain prolonged intracellular signaling and drive the sequential activation of key transcription factors such as c-Fos and nuclear factor of activated T cells 1 (NFATc1), thereby enabling progressive transcriptional and epigenetic reprogramming necessary for osteoclast differentiation [10]. For the purpose of the present review, these molecular and epigenetic events are considered primarily as time-dependent biological processes that require sustained signaling input, rather than as isolated pathway targets [11,12]. This distinction is important because it links bone biology directly to pharmacokinetic persistence and helps explain why short-lived phytochemical exposure may fail to produce durable skeletal effects despite strong in vitro activity.

Transient inhibition of osteoclastogenic signaling suppresses differentiation but does not eliminate precursor or recycled osteoclast populations, which remain poised for rapid reactivation. Consequently, such short-term interventions fail to durably alter the remodeling equilibrium, particularly under chronic inflammatory conditions that continuously drive osteoclastogenesis. As illustrated in Figure 1, Interventions producing reversible pharmacologic effects without skeletal retention, such as RANKL inhibition with denosumab, are associated with rapid loss of treatment-induced gains following withdrawal, reflecting the transient nature of their activity on bone remodeling compartments and the rebound increase in bone turnover. In contrast, agents with prolonged skeletal retention, such as bisphosphonates, may sustain antiresorptive effects beyond treatment cessation and thereby contribute to more durable preservation of bone mass [5].

Plant-derived bioactives frequently illustrate this kinetic limitation. Although many phytochemicals interact with pathways relevant to osteoclast and osteoblast regulation, isolated compounds often exhibit poor solubility, extensive metabolic conjugation, and short systemic half-lives [13].

Representative pharmacokinetic examples illustrate this limitation. Curcumin, despite extensive experimental evidence for anti-inflammatory and osteomodulatory activity, shows very low systemic availability in humans, with circulating unconjugated curcumin frequently remaining extremely low unless formulation strategies are used to improve absorption [14]. Resveratrol is efficiently absorbed but undergoes rapid phase II metabolism, so circulating exposure is dominated by sulfate and glucuronide conjugates rather than the parent compound [15]. Icariin, a flavonoid widely investigated for bone-related activity, also shows poor oral bioavailability, reported at approximately 12% in preclinical literature, which has motivated formulation strategies to improve its solubility, absorption, and systemic persistence [16]. Conversely, human apple-flavanol studies show that the whole apple matrix can reduce epicatechin bioavailability compared with an epicatechin-rich apple extract beverage, demonstrating that the transformation state can directly modify Tmax, Cmax, and systemic exposure [17]. These examples support the central premise that plant-derived skeletal efficacy depends not only on whether a molecule can regulate osteogenic or antiresorptive pathways, but also on whether its transformation state generates a pharmacokinetic profile compatible with the slow temporal dynamics of bone remodeling.

Plant-based interventions are not limited to isolated compounds but span a continuum of transformation systems, from intact plant matrices to processed extracts, purified phytochemicals, and engineered delivery platforms. Across this continuum, the composition, structure, and physicochemical form of phytochemicals are progressively modified, leading to differences in their liberation from matrices, solubilization, stability, and absorption, and thereby generating distinct systemic exposure profiles [18].

Within this context, the biological effects of plant-derived interventions should be understood not solely in terms of molecular activity, but in terms of how transformation-dependent exposure profiles align with the temporal requirements of skeletal remodeling. Different transformation states modify the timing, magnitude, and persistence of systemic exposure through distinct mechanisms, including matrix-constrained release, gastrointestinal processing, microbial biotransformation, and formulation-driven absorption enhancement. Accordingly, this review aims to adopt a transformation-state-dependent exposure framework to evaluate plant-based strategies for bone health. By integrating plant-matrix architecture, metabolic fate, and exposure persistence with the temporal dynamics of bone remodeling, we aim to clarify the sources of translational variability and establish a mechanistic basis for designing plant-derived interventions capable of achieving durable skeletal efficacy.

However, this exposure-centered interpretation should be viewed as a conceptual and mechanistic framework rather than as a validated predictive rule. Sustained exposure is likely important for skeletal relevance, but it is not sufficient by itself to guarantee therapeutic benefit. Human responses to plant-derived compounds are influenced by interindividual variability in absorption, distribution, metabolism, excretion, gut microbiota composition, gut-derived metabotypes, baseline diet, age, sex, comorbidities, medication use, and intervention standardization [19,20,21]. Clinical evidence for polyphenols and bone health remains heterogeneous, with stronger consistency often observed for mechanistic or biochemical endpoints than for long-term structural outcomes such as bone mineral density or fracture reduction [7]. Therefore, the proposed framework is intended to improve interpretation and study design, while recognizing that clinical validation, standardized preparations, dose–exposure relationships, and remodeling-relevant longitudinal endpoints remain necessary.

Importantly, the present review does not treat plant-derived compounds as a pharmacologically uniform category. Rather, it examines plant-based interventions as a continuum of transformation systems, ranging from intact matrices and extracts to isolated phytochemicals and engineered delivery platforms, each of which imposes distinct constraints on release, metabolism, systemic persistence, and ultimately skeletal relevance. Framing plant-derived interventions in this way allows bone efficacy to be interpreted not simply as a function of molecular activity, but as the outcome of alignment—or misalignment—between transformation-dependent exposure architecture and the temporal demands of skeletal remodeling, while recognizing that this framework remains hypothesis-generating and requires compound-specific, formulation-specific, and clinically validated confirmation.

## 2. Literature Search Strategy and Review Methodology

This article presents a narrative review that develops a transformation-state-dependent pharmacokinetic framework for interpreting the skeletal effects of plant-derived compounds in bone remodeling. Rather than evaluating phytochemicals solely according to intrinsic molecular activity, the review integrates current evidence regarding plant matrix architecture, gastrointestinal processing, microbial biotransformation, exposure persistence, and formulation-driven pharmacokinetic modulation within the temporal context of bone remodeling dynamics.

To identify relevant literature, a structured search of major scientific databases was conducted, including PubMed, Scopus, Web of Science, and Google Scholar. These databases were selected to capture multidisciplinary literature spanning bone biology, pharmacokinetics, phytochemistry, gastrointestinal metabolism, microbiota-related research, biomaterials, and nanodelivery systems relevant to plant-derived interventions and skeletal remodeling.

Search queries combined terminology related to bone remodeling, plant-derived compounds, pharmacokinetics, microbiota-mediated metabolism, and advanced delivery systems. Representative keywords and combinations included: “bone remodeling”, “osteoblastogenesis”, “osteoclastogenesis”, “plant-derived compounds”, “phytochemicals”, “polyphenols”, “bioavailability”, “pharmacokinetics”, “exposure architecture”, “plant matrix”, “gut–bone axis”, “microbiota-mediated metabolism”, “short-chain fatty acids”, “phytosomes”, “nanodelivery systems”, “plant-derived nanoparticles”, “extracellular vesicles”, “osteoporosis”, and “skeletal remodeling”.

The literature exploration primarily focused on studies published between 2010 and 2026, reflecting the recent expansion of research concerning gut microbiota–bone interactions, phytochemical pharmacokinetics, engineered delivery systems, and exposure-oriented interpretations of plant-based therapeutics. Earlier foundational studies were also included when directly relevant to fundamental concepts such as bone remodeling biology, plant matrix organization, polyphenol–macromolecule interactions, gastrointestinal digestion models, and exposure-dependent pharmacokinetic principles.

Publications were considered relevant when they:

Investigated the effects of plant-derived compounds, extracts, matrices, or engineered delivery systems on bone remodeling, osteoblast activity, osteoclastogenesis, or skeletal homeostasis.

Examined pharmacokinetic behavior, bioavailability, gastrointestinal transformation, microbiota-mediated metabolism, or systemic exposure profiles of phytochemicals.

Reported mechanistic, translational, or formulation-related findings relevant to exposure persistence and skeletal efficacy.

Included experimental, preclinical, clinical, or mechanistic studies published in peer-reviewed journals.

Publications were not considered when they:

Focused on unrelated pathological systems without mechanistic relevance to skeletal biology or exposure-dependent phytochemical behavior.

Lacked sufficient methodological or mechanistic description regarding pharmacokinetic, microbiota-related, or bone-associated outcomes.

Provided limited relevance to transformation-state-dependent exposure concepts or skeletal remodeling processes.

Relevant studies were identified through database searches and by manual screening of reference lists from key review articles and primary studies. Extracted information included phytochemical class, plant transformation state, formulation strategy, pharmacokinetic characteristics, microbiota-related metabolism, exposure persistence, skeletal outcomes, and implicated signaling pathways.

Because the aim of this article is to establish a conceptual and mechanistic exposure-centered framework rather than perform quantitative meta-analysis, the literature was synthesized qualitatively through thematic and mechanistic integration. Particular emphasis was placed on how transformation-dependent exposure architectures influence the temporal compatibility of plant-derived interventions with the prolonged dynamics of bone remodeling, thereby shaping translational skeletal efficacy.

## 3. Bone Remodeling as a Temporal and Exposure Filter

For plant-derived interventions, the relevance of bone remodeling lies not only in the pathways it regulates, but in the temporal constraints it imposes on exposure. Because skeletal adaptation emerges through coordinated BMU activity over extended intervals, compounds delivered in different plant-associated forms cannot be evaluated solely by target engagement or peak concentration. They must be considered in relation to whether the exposure profiles they generate can be integrated across remodeling-relevant timescales.

Bone remodeling is a tightly regulated process in which coordinated osteoclast-mediated resorption and osteoblast-driven formation renew skeletal tissue while maintaining structural integrity and mineral homeostasis [22].

**Figure 2:**
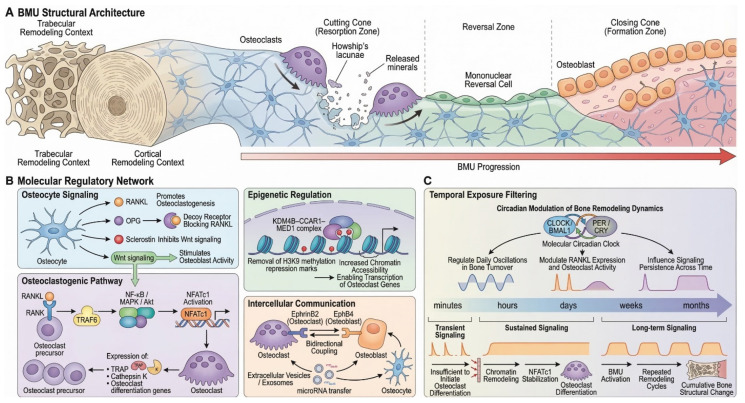
Multi-scale organization and regulation of bone remodeling within the BMU. (**A**) Spatial progression of a basic multicellular unit (BMU) showing sequential phases of osteoclast-mediated resorption (cutting cone), reversal, and osteoblast-driven formation (closing cone) during its migration through bone. (**B**) Molecular regulation of remodeling, highlighting osteocyte-mediated control (RANKL/OPG, sclerostin), osteoclastogenic signaling pathways, epigenetic regulation, and intercellular coupling between bone cells. (**C**) Temporal dynamics of remodeling, illustrating how sustained signaling and circadian regulation govern osteoclast differentiation, BMU activation, and cumulative structural adaptation over time. Generated using Nanobanana Pro.

Bone remodeling is carried out within transient, spatially and temporally coordinated BMUs, where osteoclast-mediated resorption is followed by reversal and osteoblast-driven formation phases through tightly coupled cellular interactions among osteoclasts, osteoblast-lineage cells, osteocytes, and associated stromal and vascular components [9]. As illustrated schematically in Figure 2, BMUs migrate across bone surfaces or through cortical bone while executing the sequential stages of resorption, reversal, and formation.

During the initial cutting cone phase, osteoclasts resorb old or micro-damaged bone matrix, forming resorption pits (Howship’s lacunae) and releasing mineral components and matrix-embedded growth factors, which contribute to the coupling of subsequent bone formation [23]. These resorptive events are followed by a reversal phase, during which mononuclear cells prepare the resorbed surface for subsequent bone formation. This is followed by the closing cone phase, in which osteoblasts deposit osteoid that later mineralizes to form new bone matrix. Through this coordinated progression of BMUs along the bone surface, aged or damaged tissue is continuously replaced while preserving skeletal architecture and biomechanical integrity [9].

Osteocytes regulate both osteoclast-mediated resorption and osteoblast-mediated formation through signaling molecules such as receptor activator of nuclear factor-κB ligand (RANKL), osteoprotegerin (OPG), and sclerostin [24]. Within this regulatory network, binding of RANKL to its receptor RANK activates intracellular signaling pathways involving TNF receptor-associated factor 6 (TRAF6), NF-κB, mitogen-activated protein kinase (MAPK), and Akt, which converge on activation of the transcription factor NFATc1, the principal regulator of osteoclast differentiation [25,26].

Osteoclast differentiation is also influenced by epigenetic mechanisms that regulate chromatin accessibility and transcriptional responsiveness, thereby contributing to sustained osteoclastogenic signaling and remodeling activity [27,28]. In parallel, intercellular communication pathways—including EphrinB2–EphB4 signaling and extracellular vesicle-mediated signaling—further coordinate osteoblast–osteoclast interactions within the BMU microenvironment [29,30]. For the purposes of the present review, these molecular and epigenetic mechanisms are considered primarily as time-dependent biological processes that require sustained signaling input rather than as isolated pathway targets.

Bone remodeling is additionally influenced by circadian regulatory systems involving core clock components such as CLOCK, BMAL1, PER, and CRY, which contribute to rhythmic regulation of skeletal turnover [31]. As depicted conceptually in Figure 2, these oscillatory systems integrate short-term signaling events with the slower temporal kinetics of BMU progression.

Taken together, these regulatory layers reveal bone remodeling as a multi-scale spatiotemporal system in which molecular signaling, epigenetic control, intercellular communication, and systemic timing mechanisms converge to regulate skeletal renewal. Because osteoclast differentiation and BMU activity require sustained activation of multiple signaling pathways and occur over extended timeframes, the remodeling process effectively functions as a temporal and exposure-dependent biological filter. As illustrated in Figure 2, transient signaling events lasting minutes or hours are often insufficient to initiate osteoclast differentiation, whereas sustained signals persisting across days to weeks enable chromatin remodeling, stabilization of NFATc1 transcriptional programs, and activation of BMUs. Repeated remodeling cycles occurring over months ultimately lead to cumulative structural changes in bone architecture [8]. Consequently, the skeletal system integrates molecular signals over extended time windows, allowing it to filter transient perturbations while responding robustly to persistent biological or pharmacological stimuli.

This temporal filtering property has direct implications for plant-derived interventions. Because plant matrices, extracts, isolated phytochemicals, and engineered delivery systems generate markedly different patterns of release, absorption, metabolism, and persistence, their skeletal relevance cannot be assumed to be equivalent even when they contain overlapping bioactive constituents. The critical question is therefore not only whether a plant-derived compound is osteomodulatory, but whether its transformation-dependent exposure profile is compatible with the slow kinetics of bone remodeling.

## 4. Plant Transformation States and Their Impact on Exposure Architecture

The temporal filtering properties of bone remodeling described above imply that pharmacological interventions must maintain biologically effective exposure across extended time windows to meaningfully influence skeletal turnover. This requirement is particularly relevant for plant-derived bioactives, whose pharmacokinetic behavior is strongly influenced by the structural context in which they are delivered. Plant-based interventions therefore exist along a continuum of transformation states, ranging from intact plant matrices to processed foods, crude or standardized extracts, purified phytochemicals, and engineered delivery systems. Each transformation state modifies the exposure architecture of phytochemicals by influencing their liberation from plant tissues, intestinal absorption, metabolic processing, and systemic persistence.

**Figure 3:**
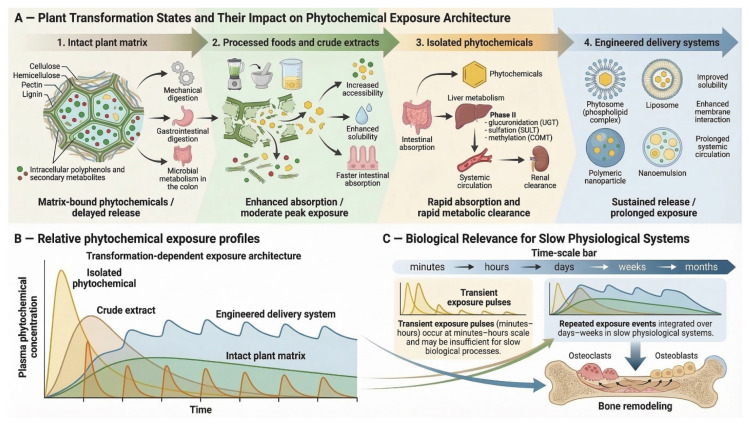
Conceptual framework of transformation-dependent phytochemical exposure and skeletal relevance. (**A**) Plant transformation states—from intact matrices to engineered delivery systems—shape phytochemical release, absorption, and metabolic fate. (**B**) These differences give rise to distinct systemic exposure profiles, ranging from sustained to transient. (**C**) The functional relevance of these profiles depends on their alignment with slow physiological processes such as bone remodeling. Generated using Nanobanana Pro. The representation of isolated phytochemicals as rapidly absorbed and rapidly cleared reflects a common pharmacokinetic pattern reported for many poorly soluble or extensively metabolized phytochemicals, but should not be interpreted as universal for all isolated plant compounds. Similarly, although engineered delivery systems may improve exposure behavior, their translational applicability remains dependent on compound-specific validation of efficacy, safety, scalability, and clinically meaningful skeletal outcomes.

These transformation-dependent exposure dynamics are conceptually summarized in Figure 3, which illustrates how structural processing of plant material progressively reshapes phytochemical availability and systemic exposure patterns across four transformation states.

Within the intact plant matrix, phytochemicals are embedded within a complex three-dimensional structural network composed primarily of cellulose, hemicellulose, pectin, lignin, and associated proteins [32,33] (Figure 3A, state 1). These macromolecular structures restrict immediate access to intracellular metabolites and frequently bind polyphenols and other secondary metabolites through hydrogen bonding, hydrophobic interactions, or ester linkages [34]. As a result, a substantial proportion of dietary polyphenols exists as matrix-bound or non-extractable forms that remain associated with plant cell-wall polymers and resist release during early stages of gastrointestinal digestion [35]. Many of these compounds reach the colon largely intact, where microbial enzymes such as esterases and glycosidases hydrolyze conjugated structures to generate smaller phenolic metabolites capable of entering systemic circulation [36,37]. Consequently, the intact plant matrix functions as a biologically buffered release system in which mechanical disruption, enzymatic digestion, and microbial fermentation progressively liberate phytochemicals along the gastrointestinal tract. Simulated digestion studies demonstrate progressive liberation of phytochemicals across oral, gastric, and intestinal phases, illustrating how the gastrointestinal tract effectively acts as an extraction environment for matrix-embedded metabolites [38]. In pharmacokinetic terms, this matrix-dependent liberation often produces delayed and relatively lower systemic peaks while extending exposure through microbiota-derived metabolites.

Processing and extraction partially dismantle this structural framework, generating processed foods and crude extracts that contain mixtures of soluble secondary metabolites with reduced matrix constraints [39,40,41] (Figure 3A, state 2).

Mechanical disruption, enzymatic treatments, thermal processing, and solvent extraction increase phytochemical accessibility and solubility, thereby accelerating intestinal uptake [42,43,44]. However, structural disruption may also expose phytochemicals to oxidation, enzymatic degradation, or chemical modification during processing [45]. Empirical comparisons illustrate how removal of the natural food matrix alters systemic exposure. In a controlled human intervention study, epicatechin delivered as an apple polyphenol beverage produced higher and earlier plasma concentrations than the same compound delivered within an apple puree matrix, demonstrating that the intact food matrix attenuates and delays systemic absorption [17]. Similarly, comparisons between raw apple, apple puree, and phenolic extracts showed that food matrices significantly reduced postprandial flavanol peaks while simultaneously altering gene-expression responses in circulating immune cells [46]. These findings demonstrate that matrix disruption not only modifies pharmacokinetic profiles but can also influence downstream biological responses.

Further along the transformation continuum, isolated phytochemicals provide chemically defined molecules that enable precise mechanistic investigation but frequently exhibit intrinsic pharmacokinetic limitations (Figure 3A, state 3). Many polyphenols and flavonoids display poor aqueous solubility, limited membrane permeability, and rapid metabolic conjugation following intestinal absorption. Phase II metabolic enzymes—including UDP-glucuronosyltransferases (UGTs), sulfotransferases (SULTs), and catechol-O-methyltransferase (COMT)—rapidly convert many polyphenols into glucuronidated, sulfated, or methylated metabolites, often reducing circulating concentrations of the parent compounds shortly after absorption [47]. These processes frequently generate relatively rapid systemic appearance followed by extensive metabolic conversion and limited persistence of the parent compounds in circulation, producing transient exposure peaks that may not persist long enough to influence biological systems that integrate signals over extended periods. Such pharmacokinetic limitations have repeatedly been identified as a major barrier to translating promising phytochemical activities observed in experimental models into consistent clinical efficacy [48].

This does not imply that isolated phytochemicals are intrinsically unsuitable for skeletal applications. Their major advantages include chemical definition, mechanistic specificity, dose standardization, and suitability for targeted pharmacokinetic optimization. The limitation is that isolation often removes matrix-dependent buffering, microbial substrate delivery, and compositional complexity that may contribute to delayed or metabolite-mediated exposure [18,49]. Therefore, the therapeutic potential of isolated phytochemicals should be assessed through exposure validation, formulation optimization, and remodeling-relevant in vivo outcomes rather than inferred from in vitro potency alone.

To address these kinetic constraints, contemporary phytopharmaceutical research increasingly employs engineered delivery systems designed to stabilize phytochemicals and extend systemic exposure (Figure 3A, state 4). Nanotechnology-based carriers—including liposomes, polymeric nanoparticles, nanoemulsions, and solid lipid nanoparticles—can encapsulate poorly soluble compounds, improving dispersion in gastrointestinal fluids, protecting them from premature degradation, and prolonging systemic circulation [50,51]. Among these approaches, phytosomes—phospholipid–phytochemical complexes—enhance membrane interaction and intestinal permeability by forming amphiphilic molecular assemblies capable of integrating into biological membranes [52,53]. Experimental studies demonstrate that phytosome formulations can markedly increase systemic exposure; for example, a silymarin phytosome increased oral bioavailability approximately sixfold relative to the free compound in preclinical models [54]. Similar nanoparticle-based strategies have been applied to phytochemicals such as curcumin, resveratrol, quercetin, and epigallocatechin gallate to improve solubility, stability, and cellular uptake [55].

The pharmacokinetic implications of these transformation states are illustrated in Figure 3B, which depicts representative systemic exposure profiles. Intact plant matrices typically produce delayed and relatively sustained exposure due to gradual release and microbial metabolism, whereas crude extracts generate moderate concentration peaks through enhanced accessibility. In contrast, isolated phytochemicals often produce sharp concentration spikes followed by rapid metabolic clearance, while engineered delivery systems aim to prolong systemic residence and smooth concentration fluctuations [56,57,58].

The biological relevance of these exposure patterns becomes particularly evident in physiological systems operating over long temporal scales. As illustrated in Figure 3C, transient exposure pulses lasting minutes or hours may be insufficient to influence slow biological programs, whereas repeated or sustained exposure events occurring over days or weeks can be integrated by long-term physiological processes such as bone remodeling [9,59]. Because BMU activity and osteoclast differentiation unfold over extended timescales, the effectiveness of plant-derived interventions ultimately depends not only on molecular activity but also on whether transformation-dependent exposure profiles align with the temporal dynamics of skeletal remodeling.

This transformation-state perspective provides a mechanistic framework for interpreting the heterogeneous outcomes reported in plant-based bone studies and offers a basis for rationally selecting phytochemical interventions whose exposure profiles are compatible with the slow kinetics of skeletal remodeling. Viewed in this way, the transformation continuum is not merely a sequence of preparative states, but a progression of exposure systems with distinct implications for skeletal efficacy. This perspective provides the basis for distinguishing natural exposure modulation by plant matrices and extracts from the kinetic fragility that often emerges after molecular isolation, and from the exposure-restoring role of engineered delivery strategies.

## 5. Natural Exposure Modulation by Plant Matrices and Extracts

Within the transformation continuum, intact plant matrices and extracts represent exposure systems in which phytochemical behavior remains strongly shaped by native structural organization or by partial structural disruption. Unlike isolated compounds or engineered delivery platforms, these forms retain varying degrees of compositional complexity and biological buffering. Their skeletal relevance, therefore, depends on how naturally modulated release, microbial transformation, and metabolic persistence influence the duration and form of systemic exposure.

**Figure 4:**
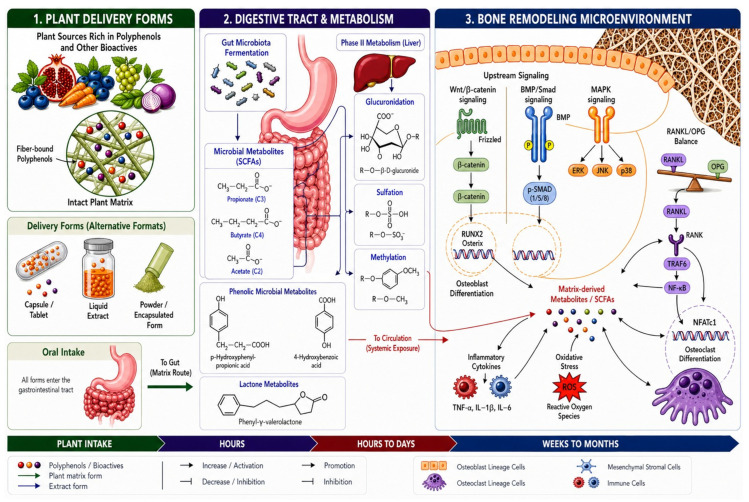
Conceptual model of matrix- and extract-driven exposure modulation in the gut–bone axis. Schematic representation of how plant delivery forms (intact matrix versus extract) generate distinct metabolic and pharmacokinetic pathways through gastrointestinal processing, liver metabolism, and microbiota-mediated transformation. Matrix-associated delivery promotes delayed and prolonged exposure via colonic metabolism, whereas extracts favor rapid absorption and transient systemic exposure. These exposure profiles differentially regulate signaling pathways in bone, influencing osteoblast and osteoclast activity and thereby shaping skeletal remodeling over extended timescales. Generated using Nanobanana Pro.

Within this context, plant matrices and extracts should be understood as distinct exposure systems that shape the timing, extent, and metabolic form in which phytochemicals interact with bone biology, as illustrated in Figure 4.

### 5.1. Matrix-Constrained Delivery and Microbiota-Mediated Bone-Relevant Exposure

In intact plant tissues, phytochemicals are embedded within structural matrices composed of cellulose, hemicellulose, pectin, and associated macromolecules. These structures limit immediate release during digestion and frequently retain substantial fractions of polyphenols in matrix-associated forms. As a result, not all ingested phytochemicals become available for absorption in the small intestine; instead, a significant portion is delivered to the colon in association with dietary fiber [35,48,60].

From a skeletal perspective, the importance of this process lies in its connection to microbiota-mediated metabolism and the gut–bone axis [61]. Polyphenols reaching the colon undergo extensive microbial transformation into smaller metabolites that are more readily absorbed and may contribute to systemic biological effects. Reviews focusing on polyphenol bioavailability emphasize that circulating metabolites often reflect microbiota-derived products rather than intact parent compounds, and that these transformations are central to understanding in vivo activity [62].

Bone-oriented literature increasingly integrates this concept. Wang and Hu highlight that gut microbial metabolism can enhance polyphenol absorption and may play an important role in osteoporosis prevention and treatment [63]. More broadly, research on the gut–bone axis identifies microbial metabolites—particularly short-chain fatty acids (SCFAs)—as regulators of bone homeostasis through effects on immune signaling, osteoclast activity, and osteoblast function [64,65].

Experimental evidence supports the relevance of matrix-dependent, microbiota-linked exposure for skeletal outcomes. In a recent animal study, dietary tart cherry provided as a complex plant matrix improved bone structure while simultaneously altering gut microbiota composition and increasing SCFAs production; suppression of the microbiota attenuated the cortical bone response, indicating that the skeletal effects were at least partly microbiota-dependent [66].

Taken together, these findings support a model in which intact plant matrices do not simply reduce or delay phytochemical absorption but instead shift exposure toward microbiota-dependent metabolic pathways. This shift may extend the functional relevance of plant-derived compounds by generating secondary metabolites and signaling interactions that persist beyond the initial absorption phase. In the context of bone remodeling—which integrates signals over extended periods—such distributed exposure patterns may be more compatible with skeletal biology than transient peaks of parent compounds.

### 5.2. Extraction-Driven Reconfiguration of Exposure and Its Skeletal Implications

Extraction and processing alter plant-derived interventions by disrupting structural constraints and increasing the accessibility of phytochemicals. As a result, extracts often contain higher concentrations of solvent-recoverable compounds and may produce greater early systemic exposure compared with intact plant matrices. However, bone-focused literature indicates that increased accessibility alone does not resolve the central limitation of phytochemical interventions—namely, insufficient exposure persistence [67].

Multiple reviews emphasize that poor bioavailability, rapid metabolism, and short systemic residence times are major barriers to the clinical translation of phytochemicals for bone health [7,68]. Specific compound-focused analyses reinforce this point. For example, icariin—a flavonoid widely studied for its osteogenic and anti-resorptive effects—exhibits low oral bioavailability and a short half-life, limiting its ability to maintain effective concentrations in vivo despite strong experimental activity [69]. Similar pharmacokinetic limitations have been described for other bone-relevant phytochemicals such as curcumin and polydatin, which undergo rapid metabolism and exhibit limited systemic persistence [70,71].

These observations highlight a critical limitation of extract-based approaches: while they may increase the amount of compound entering systemic circulation, they do not necessarily resolve the mismatch between short-lived exposure and the prolonged signaling required for bone remodeling.

In some cases, extraction may improve early bioavailability and produce measurable biological effects; in others, it may generate transient systemic peaks that remain insufficient for long-term skeletal modulation. Therefore, extracts should be interpreted as exposure-reconfigured systems whose translational value depends on phytochemical composition, dose, metabolic fate, host variability, and study duration [72].

Moreover, extraction alters not only the quantity but also the composition of phytochemical systems, typically enriching freely soluble fractions while reducing delivery of matrix-associated compounds that would otherwise reach the colon and participate in microbiota-dependent pathways [39,73].

Human evidence further underscores the importance of exposure variability. Reviews of clinical studies on polyphenols and bone consistently report heterogeneity in outcomes, which is attributed in part to differences in bioavailability, metabolism, and study design [74]. Even when bioactive compounds demonstrate clear molecular effects, their inability to maintain sustained exposure at target tissues limits their capacity to influence long-term skeletal endpoints.

These observations collectively indicate that transformation-dependent exposure behavior contributes substantially to variability across plant-based skeletal interventions. Systems promoting delayed, sustained, or metabolite-mediated exposure may be better suited to the prolonged temporal dynamics of bone remodeling than systems that produce rapid, transient systemic availability. Representative examples across transformation states are summarized in Table 1.

The studies summarized in Table 1 collectively demonstrate that plant-derived interventions must be interpreted within the context of the exposure architecture they generate, rather than solely by their intrinsic molecular activity. This distinction becomes particularly evident when comparing systems that differ in transformation state but target similar skeletal pathways. For example, fiber-rich plant matrices and polyphenol-containing foods exert their effects predominantly through microbiota-mediated biotransformation, generating metabolites such as SCFAs and secondary polyphenolic derivatives that sustain systemic signaling and are more temporally compatible with bone remodeling processes. In contrast, several isolated phytochemicals with well-established osteogenic or anti-resorptive mechanisms display limited in vivo efficacy due to rapid absorption and clearance, resulting in transient exposure profiles that fail to maintain remodeling-relevant signaling. This discrepancy is further highlighted by comparative cases in which pharmacokinetic enhancement alone markedly alters biological outcomes, as demonstrated by phospholipid-complexed systems such as curcumin phytosomes and bioenhanced formulations combining metabolic inhibitors (e.g., piperine), which increase systemic and even bone marrow exposure without fundamentally altering molecular targets.

Beyond these formulation-driven improvements, more advanced delivery systems, including plant-mediated nanoparticles and exosome-like vesicles, illustrate a further shift toward controlled and sustained exposure through enhanced cellular uptake, protection from degradation, and, in the case of vesicular systems, the delivery of regulatory cargo such as miRNAs. However, the outcomes across these systems also reveal that increasing exposure magnitude alone is insufficient; rather, efficacy depends on achieving an exposure profile that is both temporally sustained and biologically coordinated with the dynamics of osteoblast and osteoclast activity. Notably, certain direct-acting systems, including isolated phytoestrogenic compounds, demonstrate only modest skeletal effects despite clear receptor-level activity, underscoring that insufficient exposure persistence can limit translation even in the presence of mechanistic plausibility.

Taken together, these observations support a unifying interpretation in which plant transformation states define distinct pharmacokinetic regimes, each associated with characteristic patterns of release, metabolism, and systemic persistence. The variability observed across experimental and clinical outcomes can therefore be understood not as inconsistency in molecular efficacy, but as a reflection of how effectively each system aligns its exposure profile with the prolonged and cumulative nature of bone remodeling. This synthesis reinforces the central premise of the present framework, namely that the successful translation of plant-based interventions into meaningful skeletal outcomes depends on their ability to generate sustained, context-appropriate exposure architectures rather than transient or peak-driven systemic availability.

## 6. Exposure–Transformation Alignment and Implications for Skeletal Efficacy

The translational significance of plant-derived interventions becomes most apparent when the transformation continuum is interpreted as an exposure continuum with direct consequences for skeletal efficacy.

**Figure 5:**
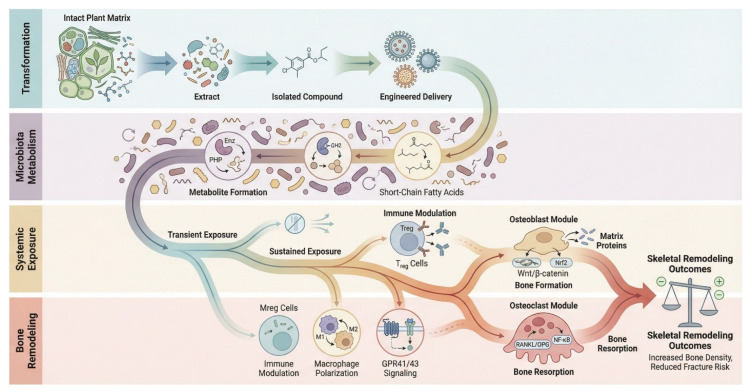
Conceptual framework of the exposure–transformation continuum and gut–bone axis. Schematic representation of how plant-derived compounds, across different transformation states, generate distinct exposure profiles through microbiota-mediated metabolism. Generated using Nanobanana Pro.

Plant-derived interventions span a continuum from intact plant matrices through crude or standardized extracts to isolated phytochemicals and engineered plant-derived delivery systems, reflecting progressive extraction and formulation processes. These forms differ not only in chemical composition but also in pharmacokinetic properties, including absorption, metabolism, and bioavailability, which determine the timing, magnitude, and persistence of systemic exposure [96,97]. Current pharmacokinetic evidence suggests that, for many dietary polyphenols, systemic biological activity is unlikely to be driven predominantly by the native parent compounds, because absorption in the small intestine is often limited or structurally constrained, whereas substantial amounts reach the colon and undergo extensive microbial catabolism to low-molecular-weight metabolites that, together with host conjugates, frequently account for a large share of circulating polyphenol-derived species [98]. Consequently, the physiological relevance of plant-derived systems is intrinsically determined by how transformation-dependent exposure profiles are generated and sustained in vivo. This integrated exposure–transformation framework, linking plant-derived inputs to microbiota-mediated metabolism, systemic pharmacokinetics, and skeletal remodeling, is illustrated in Figure 5.

Within this context, intact plant cell-wall matrices regulate the bioaccessibility of bone-active nutrients and phytochemicals by encapsulating them within a semi-permeable polysaccharide network that restricts enzymatic access and delays their release during gastrointestinal digestion. This controlled liberation modulates intestinal absorption kinetics and systemic availability, thereby influencing key molecular pathways involved in bone remodeling, including RANKL/OPG-mediated osteoclastogenesis, Wnt/β-catenin signaling in osteoblast differentiation, and oxidative stress-sensitive regulators such as NF-κB [99,100].

Gut microbiota-derived metabolites—including SCFAs, bile acids, and tryptophan derivatives—extend the pharmacokinetic profile of plant-derived substrates by enabling delayed colonic absorption and sustained systemic exposure beyond the initial intestinal phase [101]. These metabolites, which often exhibit greater bioavailability than their parent compounds, regulate bone remodeling through defined molecular pathways, including inhibition of osteoclastogenesis via GPR41/43 signaling, activation of osteoblast function, modulation of RANKL/OPG balance, and immune-mediated effects such as Treg induction and STAT6-dependent macrophage polarization. Together, these mechanisms link diet-driven microbial metabolism to prolonged systemic signaling within the gut–bone axis [102]. By comparison, isolated plant-derived compounds frequently exhibit limited aqueous solubility and undergo rapid conjugative metabolism, resulting in transient systemic exposure profiles that are dominated by short-lived metabolites rather than sustained parent compound activity [47,103].

These exposure-dependent differences are particularly relevant in bone, where plant-derived polyphenols and their metabolites regulate remodeling through coordinated effects on osteoblastogenesis, osteoclastogenesis, oxidative stress, and inflammatory signaling. Importantly, these effects are not solely determined by intrinsic molecular activity, but by the persistence and form of circulating metabolites generated through digestion and microbial transformation. Microbiota-derived metabolites can prolong systemic exposure and contribute to sustained skeletal effects, linking plant-derived inputs to bone remodeling through exposure-dependent mechanisms [7].

Specific flavonoid subclasses, particularly citrus-derived flavanones such as neohesperidin, hesperidin, and hesperetin, as well as berry-derived anthocyanins, illustrate how plant-derived compounds can exert coordinated effects on bone remodeling while remaining strongly dependent on their exposure profiles [104,105]. These compounds promote osteoblast differentiation by upregulating transcription factors such as Runx2 and Osterix, enhancing the expression of mineralization markers, including alkaline phosphatase, osteocalcin, and collagen type I, and activating signaling pathways such as Wnt/β-catenin and Small mothers against decapentaplegic (Smad)1/5/8. They also suppress osteoclastogenesis by inhibiting RANKL-mediated NF-κB, MAPK, and NFATc1 signaling. However, their in vivo skeletal efficacy is highly dependent on transformation-dependent pharmacokinetics, as these compounds are rapidly metabolized and circulate predominantly as conjugated or microbiota-derived metabolites, which determine their persistence and biological impact.

Concurrently, they suppress osteoclastogenesis by inhibiting RANKL-mediated pathways (NF-κB, MAPK, NFATc1) and shifting the RANKL/OPG balance toward reduced bone resorption. These effects are further reinforced by their antioxidant and anti-inflammatory actions, which lower reactive oxygen species (ROS) levels and pro-inflammatory cytokines (e.g., tumor necrosis factor alpha (TNF-α), interleukin (IL)-1β, IL-6), key drivers of osteoclast activation [104,105]. However, the magnitude of these osteoprotective effects is strongly modulated by pharmacokinetic factors, including low oral bioavailability, intestinal metabolism into more absorbable aglycone and conjugated metabolites, and systemic circulation levels. Notably, hesperidin exhibits low solubility and limited direct bioavailability, whereas its microbial and phase-II-derived metabolites (e.g., hesperetin conjugates) constitute the predominant circulating forms and reach higher systemic concentrations. These metabolites are considered the principal mediators of biological effects, indicating that biotransformation critically determines bioavailability and functional activity [106]. Collectively, these mechanisms contribute to increased bone formation, reduced trabecular bone loss, and improved bone mineral density in preclinical models, while highlighting pharmacokinetics as a key translational limitation and optimization target.

However, despite consistent mechanistic activity, translational outcomes remain variable, with major limitations including poor bioavailability, metabolic instability, and heterogeneity in plant preparations and study design. Clinical analyses further indicate that plant-based interventions more reliably influence biochemical markers of bone turnover than structural endpoints such as bone mineral density, suggesting that transient or insufficiently sustained exposure may limit their capacity to modulate long-term skeletal remodeling [47,107,108,109].

An additional layer of complexity arises from the gut microbiota–bone axis, whereby microbiota-mediated biotransformation of plant-derived compounds generates metabolites with distinct pharmacokinetic profiles and sustained systemic exposure. These metabolites regulate bone remodeling through integrated immune (RANKL signaling, pro- and anti-inflammatory cytokines, Treg activity), metabolic (SCFAs acting via GPR41/43 and Wnt10b), and endocrine (estrogen metabolism and parathyroid hormone (PTH) responsiveness) pathways, thereby establishing a microbiota-driven pharmacokinetic–pharmacodynamic (PK–PD) axis linking dietary inputs to skeletal homeostasis [110].

Given that plant polyphenols undergo extensive gut microbiota-mediated metabolism, their skeletal effects are mediated not only by parent compounds but also by microbiota-derived metabolites, which exhibit enhanced bioavailability and modulate bone remodeling through effects on inflammatory signaling, oxidative stress, and osteoblast–osteoclast activity [63]. This further reinforces the importance of transformation-dependent exposure architecture in determining functional outcomes. To address limitations associated with rapid metabolism and low persistence, engineered plant-derived delivery systems have been developed to modify exposure behavior by enhancing solubility, protecting against degradation, and prolonging systemic residence of phytochemical cargo [111]. However, while such systems can improve pharmacokinetic parameters, current evidence indicates that enhanced absorption alone does not guarantee improved skeletal outcomes, particularly when exposure duration remains insufficient relative to the temporal scale of bone remodeling [74,112].

Although engineered delivery systems may improve solubility, stability, systemic persistence, and exposure behavior of plant-derived compounds, their translational applicability remains dependent on compound-specific validation of efficacy, safety, scalability, regulatory feasibility, manufacturing complexity, and cost-effectiveness [113].

This highlights that the central challenge is not merely increasing systemic concentration, but achieving sustained exposure profiles compatible with slow biological processes.

Several translational limitations should nevertheless be considered when applying this exposure-centered framework to plant-derived interventions for bone health. Preclinical skeletal outcomes cannot be directly extrapolated to humans because experimental models often differ in dose, intervention duration, dietary control, and microbiota composition. In addition, plant-derived preparations vary substantially in botanical source, extraction strategy, phytochemical standardization, and formulation, while human phytochemical metabolism is highly heterogeneous due to interindividual differences in absorption, phase II metabolism, enterohepatic recycling, and microbiota-mediated biotransformation [19,20,21]. Clinical studies also evaluate different skeletal endpoints across variable timescales, ranging from biochemical markers of bone turnover to bone mineral density and fracture-related outcomes [114]. Consequently, improved pharmacokinetic exposure should be interpreted as a facilitating factor for translation rather than definitive evidence of clinical skeletal efficacy.

From this perspective, the central translational question is not which plant-derived system is intrinsically most active, but which exposure architecture is most compatible with the temporal and mechanistic requirements of bone remodeling. When considered collectively, plant transformation states define a continuum of pharmacokinetic trade-offs rather than a linear progression toward improved efficacy. Structural complexity in plant matrices and extracts can buffer and prolong exposure through delayed release and microbial metabolism, but reduces compositional precision. Isolation enhances molecular definition but introduces kinetic fragility through rapid clearance. Engineered systems can partially restore exposure persistence, yet do not fully overcome the requirement for sustained biological engagement. Thus, each transformation state represents a distinct balance between compositional complexity, metabolic stability, systemic persistence, and functional compatibility.

The value of this framework lies in its ability to reconcile part of the variability across plant-based interventions in bone-related contexts. Apparent inconsistencies in experimental and clinical findings may reflect differences in exposure architecture alongside differences in molecular potency, dose, formulation quality, host metabolism, microbiota composition, intervention duration, and study design, rather than differences in intrinsic molecular activity alone. Accordingly, the framework should be used as a research-guiding model for designing and interpreting plant-derived interventions, not as definitive evidence that any single transformation state is universally superior.

In this context, the ability of a plant-derived system to sustain biologically effective exposure over extended timeframes becomes a critical determinant of skeletal relevance. Taken together, these findings support a shift from a molecule-centered framework to a plant-system–exposure-centered framework for evaluating plant-derived strategies in bone health. The key determinant of efficacy is not only which phytochemical is present, but in which transformation state it is delivered and whether that state produces an exposure profile compatible with the temporal dynamics of skeletal remodeling. This perspective provides a mechanistic basis for interpreting heterogeneous findings across the literature and establishes a more rigorous foundation for the development of plant-based interventions with improved translational potential.

## 7. Conclusions

This review proposes a transformation-state-dependent framework in which the skeletal effects of plant-derived interventions are interpreted through the exposure architectures they generate, rather than intrinsic molecular activity alone. By integrating plant matrix structure, gastrointestinal processing, microbial biotransformation, and formulation-driven pharmacokinetics with the temporal dynamics of bone remodeling, this perspective provides a coherent explanation for part of the variability observed across experimental and clinical findings.

Across the transformation continuum, plant-derived systems produce distinct pharmacokinetic regimes. Intact matrices and microbiota-dependent pathways often favor delayed and more sustained exposure, whereas isolated phytochemicals often exhibit rapid absorption and clearance, limiting persistence. Formulation strategies, including phospholipid complexes, bioenhancers, and nano- or vesicle-based systems, can partially restore exposure by improving stability, absorption, and retention.

Representative interventions showing promising pharmacokinetic and skeletal findings include curcumin phytosome systems, resveratrol nanoformulations, icariin-loaded delivery platforms, and matrix-associated polyphenol systems that promote sustained or metabolite-mediated exposure. However, these approaches do not uniformly translate into improved skeletal outcomes, indicating that efficacy depends primarily on achieving exposure profiles compatible with the prolonged temporal requirements of bone remodeling.

The central implication of this framework is that skeletal relevance is determined by the alignment between transformation-dependent exposure profiles and the integrative nature of bone remodeling, rather than by peak concentration or compound identity alone. This perspective shifts evaluation from a molecule-centered to a system-level approach, emphasizing how plant-derived compounds are delivered, transformed, and sustained in vivo.

Nevertheless, the framework should be interpreted as a proposed conceptual and hypothesis-generating model rather than a clinically validated predictive system. Several limitations remain, including interindividual variability in metabolism and microbiota composition, heterogeneity in phytochemical preparations and formulation strategies, inconsistencies across preclinical and clinical studies, and variability in skeletal endpoints and intervention durations. In addition, enhanced pharmacokinetic exposure does not necessarily guarantee clinically meaningful skeletal benefit.

Although conceptual, this framework offers a structured basis for interpreting existing evidence and guiding future research toward exposure-informed design of plant-based interventions with improved translational potential.

Future studies should prioritize standardized phytochemical characterization, dose–exposure assessment, metabolite profiling, microbiota-associated variability, long-term remodeling-relevant endpoints, and clinically validated longitudinal studies to determine which transformation-dependent exposure strategies are most compatible with sustained skeletal modulation.

## Figures and Tables

**Table 1:** Representative plant-derived systems illustrating how transformation state and formulation strategy shape exposure architecture and skeletal outcomes. The table integrates evidence across intact plant matrices, extracts, isolated phytochemicals, and engineered delivery platforms, emphasizing how differences in release kinetics, microbial transformation, systemic persistence, and target-site exposure govern the ability of plant-derived compounds to modulate osteoblast and osteoclast activity within the temporal constraints of bone remodeling.

Plant/System	Form	Model	PK Insight (Remodeling-Relevant Exposure)	Skeletal Outcome	Mechanistic Basis	Ref
Whole Plant Matrices and Matrix-Dependent Systems						
Tart cherry (*Prunus cerasus*)	Whole plant matrix	Female C57BL/6 mice	Microbiota-dependent, SCFA-mediated secondary exposure	↑ Bone mineral density (BMD); improved trabecular and cortical bone; ↑ osteoblast activity	Gut–bone axis modulation via SCFAs	[66]
Dried plum (*Prunus domestica*)	Whole fruit, extract, fractions	Ovariectomized (OVX) mice	Matrix-dependent, microbiota-mediated exposure with SCFA contribution	↑ BMD; restored bone structure; ↓ bone loss	SCFA-mediated signaling; anti-resorptive and anabolic effects	[75]
Blueberry (*Vaccinium* spp.)	Whole-fruit powder	OVX rats; postmenopausal women	Dose-dependent microbiota-derived metabolite exposure with hormetic profile	↑ calcium retention; ↓ bone turnover markers	Phenolic metabolites regulate osteoclastogenesis and redox pathways	[76]
Dietary fiber	Whole plant matrix	Human cohort	Microbiota-dependent SCFA exposure with host variability	↑ BMD; ↓ fracture risk	SCFA-mediated inhibition of osteoclastogenesis; enhanced calcium metabolism	[77]
Soybean (*Glycine max*)	Isoflavone-enriched matrix	OVX mice	Matrix-retained exposure with composition-dependent bioavailability	↑ BMD; improved bone turnover markers	Estrogen receptor activation; modulation of osteoblast/osteoclast balance	[78]
Extracts and Partially Processed Plant Systems						
Tea polyphenols (*Camellia sinensis*)	Extract	Osteoporosis mouse model	Microbiota-mediated exposure with barrier-dependent metabolite absorption	↑ BMD; improved trabecular structure; ↓ osteoclast activity	Microbiota remodeling; intestinal barrier restoration; metabolite signaling	[79]
Mulberry polyphenols	Extract	In vitro + in vivo models	Direct exposure with sufficient bioavailability for sustained activity	↑ BMD; ↑ osteoblast activity; ↓ osteoclastogenesis	Activation of Wnt/β-catenin; PPARG inhibition; ↓ sclerostin	[80]
*Cistanche deserticola*	Glycoside/polysaccharide extracts	Osteoporosis mouse model	Composition-dependent exposure determining efficacy	↑ BMD; improved trabecular architecture	Activation of Wnt/β-catenin; ↑ BMP-2, OPG; ↓ RANKL	[81]
Ginger (*Zingiber officinale*)—10-gingerol	Extract-derived isolated phytochemical	In vitro + zebrafish	Rapid, formulation-assisted exposure with limited persistence	↓ Osteoclastogenesis; restored mineralization	Inhibition of NFATc1 and NF-κB signaling; suppression of CTSK	[82]
Isolated Phytochemicals and Purified Fractions						
Isoliquiritigenin (*Glycyrrhiza* spp.)	Isolated phytochemical	OA mouse model	Direct exposure with narrow therapeutic window and dose dependence	↓ Osteoclastogenesis; improved subchondral remodeling	Inhibition of RANKL–TRAF6 signaling; anti-angiogenic effects	[83]
Curcumin (*Curcuma longa*) + Bone marrow stromal cells (BMSCs)	Isolated phytochemical (co-system)	In vitro + OA model	Context-dependent exposure requiring cellular interaction	Improved cartilage repair; reduced OA progression	Amplification of BMSC signaling; NF-κB inhibition	[84]
*Polygonatum sibiricum* polysaccharides	Purified fraction	In vitro osteoblasts	Direct, concentration-dependent exposure with narrow effective range	↑ osteoblast differentiation; ↑ mineralization	Promotion of osteoblast maturation pathways	[85]
Licorice constituents	Isolated phytochemicals	OVX rats	Direct exposure with limited persistence	Modest improvement in bone structure	Phytoestrogenic signaling via ERα/ERβ	[86]
Barley leaf polysaccharide	Purified polysaccharide	OVX mice + in vitro	Direct exposure with sufficient systemic activity	↑ BMD; ↓ osteoclast formation	Inhibition of ERK/p38/NF-κB; suppression of NFATc1	[87]
Engineered Delivery and Exposure-Enhancing Systems						
Plant-derived phyto-nanoparticles	Engineered nano-delivery	In vitro + in vivo	Controlled and sustained release with enhanced bioavailability	↑ osteogenesis; improved bone regeneration	Activation of Wnt/BMP; modulation of RANKL/OPG	[88]
Plant-derived exosome-like nanoparticles (PDENs)	Natural nanovesicles	In vitro + in vivo	Vesicle-mediated delivery enabling protected and sustained signaling	↑ osteoblast differentiation; ↓ osteoclast activity	miRNA-mediated gene regulation; activation of BMP/Wnt/PI3K pathways	[89]
Plant-derived nanovesicles (PELNs)	Natural vesicles	In vitro + in vivo	Encapsulated delivery enabling sustained intracellular signaling	↑ osteogenesis; ↓ inflammation and osteoclast activity	Suppression of RANKL signaling; activation of osteogenic pathways	[90]
Plant-mediated phytonanoparticles	Engineered nanoparticles	In vitro + in vivo	Enhanced stability and localized retention with controlled release	↑ osteoblast activity; improved bone regeneration	Activation of Runt-related transcription factor 2 (RUNX2)/BMP/Wnt; angiogenesis promotion	[91]
Phyto-nanoparticle delivery systems	Engineered nanosystems	In vitro + in vivo	Sustained release and improved retention overcoming rapid clearance	↑ osteogenesis; ↓ osteoclast activity	Enhanced intracellular delivery; modulation of RANKL/NF-κB	[92]
Curcumin phytosome (Meriva^®^)	Phytosome formulation	Human + in vivo	Enhanced absorption and sustained exposure overcoming PK limitations	↑ bone density; improved bone parameters	Suppression of NF-κB/RANKL; promotion of osteoblast activity	[93]
Formononetin–piperine complex	Bioenhanced phytochemical system	OVX rats	Metabolism-inhibited exposure with increased half-life and bone targeting	↑ trabecular bone; improved strength	Activation of RUNX2/BMP; suppression of osteoclastogenesis	[94]
Phytosome complexes (general)	Phospholipid complexes	Human + preclinical	Improved membrane permeability and systemic exposure	↑ bone density	Enhanced cellular uptake; modulation of osteogenic pathways	[95]

↑ Symbol refers to the increase of the mentioned markers, while ↓ refers to the decrease of them.

## Data Availability

The data that support the findings of this study are available from the corresponding author upon reasonable request.

## Abbreviations

The following abbreviations are used in this manuscript:

Abbreviation	Full term
Akt	Protein kinase B
ALP	Alkaline phosphatase
AUC	Area under the curve
BMD	Bone mineral density
BMAL1	Brain and muscle ARNT-like 1
BMP	Bone morphogenetic protein
BMU	Basic multicellular unit
BMSC	Bone marrow stromal cell
CCAR1	Cell cycle and apoptosis regulator 1
CLOCK	Circadian locomotor output cycles kaput
COMT	Catechol-O-methyltransferase
COX-2	Cyclooxygenase-2
CRY	Cryptochrome
CTSK	Cathepsin K
CYP	Cytochrome P450
DNA	Deoxyribonucleic acid
EphB4	EPH receptor B4
ER	Estrogen receptor
EVs	Extracellular vesicles
GPR41/43	G protein-coupled receptors 41 and 43
IL	Interleukin
Jmjd3	Jumonji domain-containing protein 3
KDM4B	Lysine demethylase 4B
MAPK	Mitogen-activated protein kinase
MED1	Mediator complex subunit 1
NFATc1	Nuclear factor of activated T cells 1
NF-κB	Nuclear factor kappa B
OA	Osteoarthritis
OPG	Osteoprotegerin
OVX	Ovariectomized
PANoptosis	Pyroptosis, apoptosis, and necroptosis
PDENs	Plant-derived exosome-like nanoparticles
PELNs	Plant exosome-like nanovesicles
PER	Period circadian regulator
PI3K	Phosphoinositide 3-kinase
PK	Pharmacokinetic
PK–PD	Pharmacokinetic–pharmacodynamic
PPARγ	Peroxisome proliferator-activated receptor gamma
RANK	Receptor activator of nuclear factor kappa B
RANKL	Receptor activator of nuclear factor kappa B ligand
RNA	Ribonucleic acid
ROS	Reactive oxygen species
SCFAs	Short-chain fatty acids
SEM	Standard error of the mean
Smad	Small mothers against decapentaplegic
SMI	Structure model index
SOST	Sclerostin
STAT6	Signal transducer and activator of transcription 6
SULT	Sulfotransferase
TGF-β	Transforming growth factor beta
TNF-α	Tumor necrosis factor alpha
TRAF6	TNF receptor-associated factor 6
TRAP	Tartrate-resistant acid phosphatase
UGT	UDP-glucuronosyltransferase
VEGF	Vascular endothelial growth factor
Wnt	Wingless/integrated

## References

[1] Adejuyigbe B., Kallini J., Chiou D., Kallini J.R. (2023). Osteoporosis: Molecular pathology, diagnostics, and therapeutics. Int. J. Mol. Sci..

[2] Zhang X., Liang Y., Zhang F., Liu X. (2025). Osteoporosis: Molecular pathogenesis and therapeutic interventions. Mol. Biomed..

[3] Gregson C.L., Armstrong D.J., Bowden J., Cooper C., Edwards J., Gittoes N.J.L., Harvey N., Kanis J., Leyland S., Low R. (2022). Correction: UK clinical guideline for the prevention and treatment of osteoporosis. Arch. Osteoporos..

[4] Gregson C.L., Armstrong D.J., Avgerinou C., Bowden J., Cooper C., Douglas L., Edwards J., Gittoes N.J.L., Harvey N.C., Kanis J.A. (2025). The 2024 UK clinical guideline for the prevention and treatment of osteoporosis. Arch. Osteoporos..

[5] Kumar S., Wang M., Kim A.S., Center J.R., McDonald M.M., Girgis C.M. (2025). Denosumab discontinuation in the clinic: Implications of rebound bone turnover and emerging strategies to prevent bone loss and fractures. J. Bone Miner. Res..

[6] Ouyang X., Ma Q., Zhou C., Tang J., Li M., Qing J., Lei X., Huang D., Liu H., Zhang G. (2025). Natural bioactive products in the regulation of bone metabolism and regeneration. Front. Pharmacol..

[7] Perrone P., De Rosa C., D’Angelo S. (2025). Polyphenols and bone health: A comprehensive review of their role in osteoporosis prevention and treatment. Molecules.

[8] Kenkre J.S., Bassett J.H. (2018). The bone remodelling cycle. Ann. Clin. Biochem..

[9] Bolamperti S., Villa I., Rubinacci A. (2022). Bone remodeling: An operational process ensuring survival and bone mechanical competence. Bone Res..

[10] Zhu S., Yan M.-Q., Masson A., Chen W., Li Y.-P. (2026). Cell signaling and transcriptional regulation of osteoclast lineage commitment, differentiation, bone resorption and diseases. Cell Discov..

[11] Kurotaki D., Yoshida H., Tamura T. (2020). Epigenetic and transcriptional regulation of osteoclast differentiation. Bone.

[12] Appelman-Dijkstra N.M., Oei H.L.D., Vlug A.G., Winter E.M. (2022). The effect of osteoporosis treatment on bone mass. Best Pract. Res. Clin. Endocrinol. Metab..

[13] Sahraeian S., Rashidinejad A., Golmakani M.-T. (2024). Recent advances in the conjugation approaches for enhancing the bioavailability of polyphenols. Food Hydrocoll..

[14] Kroon M.A.G.M., van Laarhoven H.W.M., Swart E.L., van Tellingen O., Kemper E.M. (2025). A pharmacokinetic study and critical reappraisal of curcumin formulations enhancing bioavailability. iScience.

[15] Kapetanovic I.M., Muzzio M., Huang Z., Thompson T.N., McCormick D.L. (2011). Pharmacokinetics, oral bioavailability, and metabolic profile of resveratrol and its dimethylether analog, pterostilbene, in rats. Cancer Chemother. Pharmacol..

[16] Szabó R., Rácz C.P., Dulf F.V. (2022). Bioavailability Improvement Strategies for Icariin and Its Derivates: A Review. Int. J. Mol. Sci..

[17] Hollands W.J., Hart D.J., Dainty J.R., Hasselwander O., Tiihonen K., Wood R., Kroon P.A. (2013). Bioavailability of epicatechin and effects on nitric oxide metabolites of an apple flavanol-rich extract supplemented beverage compared to a whole apple puree: A randomized, placebo-controlled, crossover trial. Mol. Nutr. Food Res..

[18] Hu Y., Lin Q., Zhao H., Li X., Sang S., McClements D.J., Long J., Jin Z., Wang J., Qiu C. (2023). Bioaccessibility and bioavailability of phytochemicals: Influencing factors, improvements, and evaluations. Food Hydrocoll..

[19] Favari C., Rinaldi de Alvarenga J.F., Sánchez-Martínez L., Tosi N., Mignogna C., Cremonini E., Manach C., Bresciani L., Del Rio D., Mena P. (2024). Factors driving the inter-individual variability in the metabolism and bioavailability of (poly)phenolic metabolites: A systematic review of human studies. Redox Biol..

[20] Morand C. (2024). How to better consider and understand interindividual variability in response to polyphenols in clinical trials. Front. Nutr..

[21] Hu J., Mesnage R., Tuohy K., Heiss C., Rodriguez-Mateos A. (2024). (Poly) phenol-related gut metabotypes and human health: An update. Food Funct..

[22] Hu H.-T., Zhang Z.-Y., Luo Z.-X., Ti H.-B., Wu J.-J., Nie H., Yuan Z.-D., Wu X., Zhang K.-Y., Shi S.-W. (2025). Emerging regulated cell death mechanisms in bone remodeling: Decoding ferroptosis, cuproptosis, disulfidptosis, and PANoptosis as therapeutic targets for skeletal disorders. Cell Death Discov..

[23] Daponte V., Henke K., Drissi H. (2024). Current perspectives on the multiple roles of osteoclasts: Mechanisms of osteoclast–osteoblast communication and potential clinical implications. Elife.

[24] Pajevic P.D., Krause D.S. (2019). Osteocyte regulation of bone and blood. Bone.

[25] Anzai M., Watanabe-Takahashi M., Kawabata H., Masuda Y., Ikegami A., Okuda Y., Waku T., Sakurai H., Nishikawa K., Inoue J.-i. (2025). Clustered peptide regulating the multivalent interaction between RANK and TRAF6 inhibits osteoclastogenesis by fine-tuning signals. Commun. Biol..

[26] Ayyasamy R., Fan S., Czernik P., Lecka-Czernik B., Chattopadhyay S., Chakravarti R. (2024). 14-3-3ζ suppresses RANKL signaling by destabilizing TRAF6. J. Biol. Chem..

[27] Yi S.-J., Jang Y.-J., Kim H.-J., Lee K., Lee H., Kim Y., Kim J., Hwang S.Y., Song J.S., Okada H. (2021). The KDM4B–CCAR1–MED1 axis is a critical regulator of osteoclast differentiation and bone homeostasis. Bone Res..

[28] Astleford K., Campbell E., Norton A., Mansky K.C. (2020). Epigenetic regulators involved in osteoclast differentiation. Int. J. Mol. Sci..

[29] Wang R., Luo H., Yang D., Yu B., Guo J., Shao L., Okamura H., Qiu L. (2023). Osteoblast Jmjd3 regulates osteoclastogenesis via EphB4 and RANKL signalling. Oral Dis..

[30] Yuan F.-L., Wu Q.-y., Miao Z.-N., Xu M.-H., Xu R.-S., Jiang D.-L., Ye J.-X., Chen F.-h., Zhao M.-D., Wang H.-j. (2018). Osteoclast-derived extracellular vesicles: Novel regulators of osteoclastogenesis and osteoclast–osteoblasts communication in bone remodeling. Front. Physiol..

[31] Kikyo N. (2024). Circadian regulation of bone remodeling. Int. J. Mol. Sci..

[32] Ayala Soto F.E., Serna Saldívar S.O. (2020). Architecture, structure and chemistry of plant cell walls and their constituents. Science and Technology of Fibers in Food Systems.

[33] Cosgrove D.J. (2024). Structure and growth of plant cell walls. Nat. Rev. Mol. Cell Biol..

[34] Le Bourvellec C., Renard C.M. (2012). Interactions between polyphenols and macromolecules: Quantification methods and mechanisms. Crit. Rev. Food Sci. Nutr..

[35] Pérez-Jiménez J., Díaz-Rubio M.E., Saura-Calixto F. (2013). Non-extractable polyphenols, a major dietary antioxidant: Occurrence, metabolic fate and health effects. Nutr. Res. Rev..

[36] Leonard W., Zhang P., Ying D., Fang Z. (2021). Hydroxycinnamic acids on gut microbiota and health. Compr. Rev. Food Sci. Food Saf..

[37] Mahdi L., Graziani A., Baffy G., Mitten E.K., Portincasa P., Khalil M. (2025). Unlocking polyphenol efficacy: The role of gut microbiota in modulating bioavailability and health effects. Nutrients.

[38] Tagliazucchi D., Verzelloni E., Conte A. (2012). The first tract of alimentary canal as an extractor. Release of phytochemicals from solid food matrices during simulated digestion. J. Food Biochem..

[39] Yuan S., Zhao W., Wang Y., Dong H., Song K., Shi D. (2026). Mechanism-Driven Green Extraction of Plant Polyphenols: From Molecular Interactions to Process Integration and Intelligent Optimization. Plants.

[40] Cortés-Ferré H., Arredondo-Ochoa T., Gaytán-Martínez M. (2025). Polysaccharides-polyphenolic interactions: Formation, functionality and applications. Trends Food Sci. Technol..

[41] Al-Kafaween M.A., Alwahsh M., Mohd Hilmi A.B., Abulebdah D.H. (2023). Physicochemical characteristics and bioactive compounds of different types of honey and their biological and therapeutic properties: A comprehensive review. Antibiotics.

[42] Polia F., Pastor-Belda M., Martínez-Blázquez A., Horcajada M.-N., Tomás-Barberán F.A., García-Villalba R. (2022). Technological and biotechnological processes to enhance the bioavailability of dietary (poly) phenols in humans. J. Agric. Food Chem..

[43] Dai J., Mumper R.J. (2010). Plant phenolics: Extraction, analysis and their antioxidant and anticancer properties. Molecules.

[44] Brodkorb A., Egger L., Alminger M., Alvito P., Assunção R., Ballance S., Bohn T., Bourlieu-Lacanal C., Boutrou R., Carrière F. (2019). INFOGEST static in vitro simulation of gastrointestinal food digestion. Nat. Protoc..

[45] Xiao J. (2022). Recent advances on the stability of dietary polyphenols. Efood.

[46] Monfoulet L.-E., Buffière C., Istas G., Dufour C., Le Bourvellec C., Mercier S., Bayle D., Boby C., Remond D., Borel P. (2020). Effects of the apple matrix on the postprandial bioavailability of flavan-3-ols and nutrigenomic response of apple polyphenols in minipigs challenged with a high fat meal. Food Funct..

[47] Hoda M., Hemaiswarya S., Doble M. (2019). Pharmacokinetics and pharmacodynamics of polyphenols. Role of Phenolic Phytochemicals in Diabetes Management: Phenolic Phytochemicals and Diabetes.

[48] Bohn T., McDougall G.J., Alegría A., Alminger M., Arrigoni E., Aura A.M., Brito C., Cilla A., El S.N., Karakaya S. (2015). Mind the gap—Deficits in our knowledge of aspects impacting the bioavailability of phytochemicals and their metabolites—A position paper focusing on carotenoids and polyphenols. Mol. Nutr. Food Res..

[49] Kanimozhi N., Sukumar M. (2025). Harnessing probiotic fermentation to enhance the bioavailability and health impact of dietary phytochemicals. Food Wellness.

[50] Burdușel A.-C., Andronescu E. (2022). Lipid nanoparticles and liposomes for bone diseases treatment. Biomedicines.

[51] Yang B., Dong Y., Wang F., Zhang Y. (2020). Nanoformulations to enhance the bioavailability and physiological functions of polyphenols. Molecules.

[52] Kumbhar S., Jagdale V., Bhatia M. (2024). Phytosomes: A Cutting-Edge Platform for Phytochemicals Delivery by Enhancing Bioavailability. Jordan J. Pharm. Sci..

[53] Belcaro G., Cesarone M.R., Dugall M., Pellegrini L., Ledda A., Grossi M.G., Togni S., Appendino G. (2010). Efficacy and safety of Meriva^®^, a curcumin-phosphatidylcholine complex, during extended administration in osteoarthritis patients. Altern. Med. Rev..

[54] Shriram R.G., Moin A., Alotaibi H.F., Khafagy E.-S., Al Saqr A., Abu Lila A.S., Charyulu R.N. (2022). Phytosomes as a plausible nano-delivery system for enhanced oral bioavailability and improved hepatoprotective activity of silymarin. Pharmaceuticals.

[55] Goktas Z., Zu Y., Abbasi M., Galyean S., Wu D., Fan Z., Wang S. (2020). Recent advances in nanoencapsulation of phytochemicals to combat obesity and its comorbidities. J. Agric. Food Chem..

[56] Kumar A., P N., Kumar M., Jose A., Tomer V., Oz E., Proestos C., Zeng M., Elobeid T., K S. (2023). Major phytochemicals: Recent advances in health benefits and extraction method. Molecules.

[57] Sun S., Yu Y., Jo Y., Han J.H., Xue Y., Cho M., Bae S.-J., Ryu D., Park W., Ha K.-T. (2025). Impact of extraction techniques on phytochemical composition and bioactivity of natural product mixtures. Front. Pharmacol..

[58] Mungwari C.P., King’ondu C.K., Sigauke P., Obadele B.A. (2025). Conventional and modern techniques for bioactive compounds recovery from plants. Sci. Afr..

[59] Takegahara N., Kim H., Choi Y. (2024). Unraveling the intricacies of osteoclast differentiation and maturation: Insight into novel therapeutic strategies for bone-destructive diseases. Exp. Mol. Med..

[60] Arranz S., Silván J.M., Saura-Calixto F. (2010). Nonextractable polyphenols, usually ignored, are the major part of dietary polyphenols: A study on the Spanish diet. Mol. Nutr. Food Res..

[61] Xu X., Jia X., Mo L., Liu C., Zheng L., Yuan Q., Zhou X. (2017). Intestinal microbiota: A potential target for the treatment of postmenopausal osteoporosis. Bone Res..

[62] Scott M.B., Styring A.K., McCullagh J.S.O. (2022). Polyphenols: Bioavailability, Microbiome Interactions and Cellular Effects on Health in Humans and Animals. Pathogens.

[63] Wang K., Hu S. (2023). The synergistic effects of polyphenols and intestinal microbiota on osteoporosis. Front. Immunol..

[64] Zaiss M.M., Jones R.M., Schett G., Pacifici R. (2019). The gut-bone axis: How bacterial metabolites bridge the distance. J. Clin. Investig..

[65] Yan Q., Cai L., Guo W. (2022). New Advances in Improving Bone Health Based on Specific Gut Microbiota. Front. Cell. Infect. Microbiol..

[66] Adedigba P., Ice J.A., Alake S.E., Hatter B., Islam P., Ford Versypt A.N., Knotts T.A., Ritchey J., Lucas E.A., Smith B.J. (2025). Dietary Tart Cherry and Fructooligosaccharides Promote Bone Health via the Gut Microbiota and Increased Bone Formation. Nutrients.

[67] Russell R.G.G. (2011). Bisphosphonates: The first 40years. Bone.

[68] Scarpa E.-S., Antonelli A., Balercia G., Sabatelli S., Maggi F., Caprioli G., Giacchetti G., Micucci M. (2024). Antioxidant, Anti-Inflammatory, Anti-Diabetic, and Pro-Osteogenic Activities of Polyphenols for the Treatment of Two Different Chronic Diseases: Type 2 Diabetes Mellitus and Osteoporosis. Biomolecules.

[69] Hu W., Si Y., Xie X., Xu J. (2025). Research Progress on Icariin Promoting Bone Injury Repair and Regeneration. Pharmaceuticals.

[70] Khezri K., Saeedi M., Mohammadamini H., Zakaryaei A.S. (2021). A comprehensive review of the therapeutic potential of curcumin nanoformulations. Phytother. Res..

[71] Zhang Z., Sun Z., Jia R., Jiang D., Xu Z., Zhang Y., Wu Y.-Q., Wang X. (2024). Protective effects of polydatin against bone and joint disorders: The in vitro and in vivo evidence so far. Nutr. Res. Rev..

[72] Jawad M., Talcott S.T., Brannan R.G., Hillman A.R. (2026). Metabolic fate of montmorency tart cherry (Prunus cerasus L.) polyphenols and their association with short-chain fatty acids in humans: A secondary analysis. Food Biosci..

[73] Tian X.-Y., Bai J.-W., Fang Q., Wang M., Wu X., Aheto J.H. (2025). Polyphenols in Modern Nutrition: Green Extraction Technologies, Encapsulation, and Promissory Applications. Food Bioprocess Technol..

[74] Austermann K., Baecker N., Stehle P., Heer M. (2019). Putative Effects of Nutritive Polyphenols on Bone Metabolism In Vivo—Evidence from Human Studies. Nutrients.

[75] Smith B.J., Hatter B., Washburn K., Graef-Downard J., Ojo B.A., El-Rassi G.D., Cichewicz R.H., Payton M., Lucas E.A. (2022). Dried Plum’s Polyphenolic Compounds and Carbohydrates Contribute to Its Osteoprotective Effects and Exhibit Prebiotic Activity in Estrogen Deficient C57BL/6 Mice. Nutrients.

[76] Hodges J.K., Maiz M., Cao S., Lachcik P.J., Peacock M., McCabe G.P., McCabe L.D., Cladis D.P., Jackson G.S., Ferruzzi M.G. (2023). Moderate consumption of freeze-dried blueberry powder increased net bone calcium retention compared with no treatment in healthy postmenopausal women: A randomized crossover trial. Am. J. Clin. Nutr..

[77] Zhou T., Wang M., Ma H., Li X., Heianza Y., Qi L. (2021). Dietary fiber, genetic variations of gut microbiota-derived short-chain fatty acids, and bone health in UK biobank. J. Clin. Endocrinol. Metab..

[78] Kim S.M., Lee H.S., Jung J.I., Lim S.-M., Lim J.H., Ha W.-H., Jeon C.L., Lee J.-Y., Kim E.J. (2018). Effect of isoflavone-enriched whole soy milk powder supplementation on bone metabolism in ovariectomized mice. Nutr. Res. Pract..

[79] Wen X., Wu P., Li F., Pi G. (2024). Study on the relationship between tea polyphenols alleviating osteoporosis and the changes of microorganism-metabolite-intestinal barrier. Microb. Pathog..

[80] Xue L., Li J., Sun L., Liu T., Lam B., Xing K., Liang B., Hu J., Zheng Z., Yang Y. (2026). Mulberry polyphenols (ABRU) promote bone formation and alleviate bone loss via dual regulation of bone metabolism. J. Ethnopharmacol..

[81] Wang F., Tu P., Zeng K., Jiang Y. (2021). Total glycosides and polysaccharides of Cistanche deserticola prevent osteoporosis by activating Wnt/β-catenin signaling pathway in SAMP6 mice. J. Ethnopharmacol..

[82] Zang L., Kagotani K., Nakayama H., Bhagat J., Fujimoto Y., Hayashi A., Sono R., Katsuzaki H., Nishimura N., Shimada Y. (2021). 10-gingerol suppresses osteoclastogenesis in RAW264. 7 cells and zebrafish osteoporotic scales. Front. Cell Dev. Biol..

[83] Ji B., Zhang Z., Guo W., Ma H., Xu B., Mu W., Amat A., Cao L. (2018). Isoliquiritigenin blunts osteoarthritis by inhibition of bone resorption and angiogenesis in subchondral bone. Sci. Rep..

[84] Zhang R., Zhang Q., Zou Z., Li Z., Jin M., An J., Li H., Ma J. (2021). Curcumin supplementation enhances bone marrow mesenchymal stem cells to promote the anabolism of articular chondrocytes and cartilage repair. Cell Transplant..

[85] Liu J., Li T., Chen H., Yu Q., Yan C. (2021). Structural characterization and osteogenic activity in vitro of novel polysaccharides from the rhizome of Polygonatum sibiricum. Food Funct..

[86] Klasik-Ciszewska S., Kaczmarczyk-Sedlak I., Wojnar W. (2016). Effect of glabridin and glycyrrhizic acid on histomorphometric parameters of bones in ovariectomized rats. Acta Pol. Pharm..

[87] Hwang Y.-H., Ha H., Kim R., Cho C.-W., Song Y.-R., Hong H.-D., Kim T. (2019). Protective effects of a polysaccharide BLE0 isolated from barley leaf on bone loss in ovariectomized mice. Int. J. Biol. Macromol..

[88] Joseph B., Anil S., Suresh N., Waltimo T., Haidar Z.S. (2024). Phyto-Nanoparticles in Osteogenesis. Innovation in Osteogenesis Research.

[89] Yuan S., He D.-W., Zhou X.-J., Jiao H.-T., Gao Y., Li C. (2026). Greening Bone Healing: The Emerging Role of Plant-Derived Exosome-Like Nanoparticles in Osteoporosis and Osteoarthritis Therapy. Int. J. Nanomed..

[90] Xia X., Zhu J., Xu X., Wang W., Zhao R., Wu K., Huang H., Qian Y., Luo Z., Xu F. (2025). Current progress of plant-derived exosome-like nanovesicles on the regulation of osteoporosis and osteoarthritis. Ann. Med..

[91] Suresh N., Thomas N.G., Mauramo M., Waltimo T., Sorsa T., Anil S. (2025). Phytonanoparticles as novel drug carriers for enhanced osteogenesis and osseointegration. Discov. Nano.

[92] Dayanandan A.P., Cho W.J., Kang H., Bello A.B., Kim B.J., Arai Y., Lee S.-H. (2023). Emerging nano-scale delivery systems for the treatment of osteoporosis. Biomater. Res..

[93] Riva A., Franceschi F., Togni S., Eggenhoffner R., Giacomelli L. (2017). Health benefits of curcumin and curcumin phytosome in bone density disorders. JSM Bone Marrow Res..

[94] Agarwal A., Dadge S.D., Garg R., Chauhan D., Katekar R., Maity D., Vishwakarma S.K., Rathaur S., Yadav S., Gayen J.R. (2025). Formononetin–piperine–phospholipid complex: Enhancement of anti-osteoporotic activity and bioavailability in bone marrow in rats. Future J. Pharm. Sci..

[95] Reddy S., Sharma A. (2022). Characterization, properties and formulation of Phytosomes. Int. J. Multidiscip. Trends.

[96] Amin A., Akhtar M.S., Khalil A.A.K., Ali S., Zaman W. (2026). Natural products in medicinal chemistry: Targeting inflammatory pathways with plant-derived compounds. Med. Chem. Res..

[97] Bitwell C., Indra S.S., Luke C., Kakoma M.K. (2023). A review of modern and conventional extraction techniques and their applications for extracting phytochemicals from plants. Sci. Afr..

[98] Williamson G., Clifford M.N. (2017). Role of the small intestine, colon and microbiota in determining the metabolic fate of polyphenols. Biochem. Pharmacol..

[99] Holland C., Ryden P., Edwards C.H., Grundy M.M.L. (2020). Plant Cell Walls: Impact on Nutrient Bioaccessibility and Digestibility. Foods.

[100] Ticinesi A., Siniscalchi C., Meschi T., Nouvenne A. (2025). Gut microbiome and bone health: Update on mechanisms, clinical correlations, and possible treatment strategies. Osteoporos. Int..

[101] Hwang D., Chong E., Li Y., Li Y., Roh K. (2025). Deciphering the gut microbiome’s metabolic code: Pathways to bone health and novel therapeutic avenues. Front. Endocrinol..

[102] Lucas S., Omata Y., Hofmann J., Böttcher M., Iljazovic A., Sarter K., Albrecht O., Schulz O., Krishnacoumar B., Krönke G. (2018). Short-chain fatty acids regulate systemic bone mass and protect from pathological bone loss. Nat. Commun..

[103] Aatif M. (2023). Current Understanding of Polyphenols to Enhance Bioavailability for Better Therapies. Biomedicines.

[104] Ortiz A.D., Fideles S.O., Reis C.H., Bellini M.Z., Pereira E.D., Pilon J.P., de Marchi M.Â., Detregiachi C.R., Flato U.A., Trazzi B.F. (2022). Therapeutic Effects of Citrus Flavonoids Neohesperidin, Hesperidin and Its Aglycone, Hesperetin on Bone Health. Biomolecules.

[105] Perna S., Cornelli N., Acharya A., Patta E., Mazzola G., Rondanelli M., Riso P. (2026). Citrus polyphenols for bone health: A systematic review of experimental and human studies. J. Funct. Foods.

[106] Ávila-Gálvez M.Á., Giménez-Bastida J.A., González-Sarrías A., Espín J.C. (2021). New Insights into the Metabolism of the Flavanones Eriocitrin and Hesperidin: A Comparative Human Pharmacokinetic Study. Antioxidants.

[107] Brown J.P., Don-Wauchope A., Douville P., Albert C., Vasikaran S.D. (2022). Current use of bone turnover markers in the management of osteoporosis. Clin. Biochem..

[108] Inpan R., Dukaew N., Na Takuathung M., Teekachunhatean S., Koonrungsesomboon N. (2024). Effects of isoflavone interventions on bone turnover markers and factors regulating bone metabolism in postmenopausal women: A systematic review and meta-analysis of randomized controlled trials. Arch. Osteoporos..

[109] Karimi S.M., Abbaspour F., Tabatabaei-Malazy O., Qolami H., Fayyaz F., Ebrahimi Fana S., Rahimi R., Salari P., Larijani B. (2025). Impact of medicinal plants on bone health; a systematic review and meta-analysis of clinical studies. J. Agric. Food Res..

[110] Lyu Z., Hu Y., Guo Y., Liu D. (2023). Modulation of bone remodeling by the gut microbiota: A new therapy for osteoporosis. Bone Res..

[111] Saran V., Jeeva A., Syamala G. (2026). Nanotechnology-enabled delivery systems to improve the bioavailability and efficacy of polyphenolic compounds. Pharmacol. Res.-Nat. Prod..

[112] Salvio G., Ciarloni A., Gianfelice C., Lacchè F., Sabatelli S., Giacchetti G., Balercia G. (2023). The Effects of Polyphenols on Bone Metabolism in Postmenopausal Women: Systematic Review and Meta-Analysis of Randomized Control Trials. Antioxidants.

[113] Patra J.K., Das G., Fraceto L.F., Campos E.V.R., Rodriguez-Torres M.d.P., Acosta-Torres L.S., Diaz-Torres L.A., Grillo R., Swamy M.K., Sharma S. (2018). Nano based drug delivery systems: Recent developments and future prospects. J. Nanobiotechnology.

[114] Eastell R., Pigott T., Gossiel F., Naylor K.E., Walsh J.S., Peel N.F.A. (2018). DIAGNOSIS OF ENDOCRINE DISEASE: Bone turnover markers: Are they clinically useful?. Eur. J. Endocrinol..

